# Study on the Application of an Ultra-High-Frequency Fractal Antenna to Partial Discharge Detection in Switchgears

**DOI:** 10.3390/s131217362

**Published:** 2013-12-16

**Authors:** Chenguo Yao, Pan Chen, Congjian Huang, Yu Chen, Panpan Qiao

**Affiliations:** 1 State Key Laboratory of Power Transmission Equipment & System Security and New Technology, Chongqing University, Chongqing 400044, China; E-Mails: yaochenguo@cqu.edu.cn (C.Y.); 20111102011@cqu.edu.cn (P.C.); huangcongjian@cqu.edu.cn (C.H.); 20072221@cqu.edu.cn (P.Q.); 2 Jiang Bei Power Supply Bureau, Chongqing 401147, China

**Keywords:** UHF, PD, switchgear, UHF-sensor, FDTD, propagation characteristics, installation position of sensor

## Abstract

The ultra-high-frequency (UHF) method is used to analyze the insulation condition of electric equipment by detecting the UHF electromagnetic (EM) waves excited by partial discharge (PD). As part of the UHF detection system, the UHF sensor determines the detection system performance in signal extraction and recognition. In this paper, a UHF antenna sensor with the fractal structure for PD detection in switchgears was designed by means of modeling, simulation and optimization. This sensor, with a flat-plate structure, had two resonance frequencies of 583 MHz and 732 MHz. In the laboratory, four kinds of insulation defect models were positioned in the testing switchgear for typical PD tests. The results show that the sensor could reproduce the electromagnetic waves well. Furthermore, to optimize the installation position of the inner sensor for achieving best detection performance, the precise simulation model of switchgear was developed to study the propagation characteristics of UHF signals in switchgear by finite-difference time-domain (FDTD) method. According to the results of simulation and verification test, the sensor should be positioned at the right side of bottom plate in the front cabinet. This research established the foundation for the further study on the application of UHF technique in switchgear PD online detection.

## 1. Introduction

Insulation defects, such as protrusions, bad contacts and free metallic particles, may trigger partial discharges (PDs) in high-voltage equipment, and in most occasions, the existence of PD in equipment is treated as the indicator of insulation integrity degeneration [1]. Although PD has little influence on the insulation strength, without appropriate corrective measures it can come to be the cause of insulation flashovers or breakdowns [2]. Therefore, the detection of PD has become one of the most significant insulation condition assessment methods [3]. High-voltage switchgears which directly provide power supply to distribution networks and consumers have been widely used in the power system. Great economic costs have resulted from blackouts due to switchgear equipment faults. Furthermore, switchgears have a quite complex internal structure, many parts of differing physical qualities and small internal space. As a result, these devices are prone to be affected by partial discharges.

Currently, the main means of PD detection for switchgears involve ultrasonic detection [4–6], radio frequency (RF) detection [7], transient earth voltage detection [8] and UHF detection [9,10]. In comparison, the UHF method features a great anti-interference capability, high sensitivity and broad application. As part of UHF detection system, the performance of the UHF sensor directly conditions the whole detection system performance in signal extraction and recognition. Basically, UHF antenna sensors can be broken down into two main categories: internal and external. The electromagnetic waves generated by PD are shielded by the steel casing of the switchgear, but a small amount of electromagnetic waves still radiates outside the box from joints and outgoing line ports. These radio waves decay severely in the air. For this reason, the sensitivity of the UHF method is not very high when the UHF sensor is installed outside the cabinet. However, if an inner sensor is adopted, not only will the detection sensitivity be increased, but the outer electromagnetic interference will also be avoided. Most UHF antenna sensors described in the related research are external ones. Furthermore, existing UHF inner sensors are designed for some specific devices such as transformers and gas insulated switchgear (GIS) [11,12]. A special inner sensor designed for switchgear hasn't been developed yet. Besides, there is a particularly close relationship between the location of the UHF sensor and the sensitivity of this method. The object of study in the UHF technique is electromagnetic waves. Because of the limited space and the complex structure of the switchgear, its components affect the propagation of the radio waves differently, which makes it hard to determine the best place to install the sensor [8]. Intensive study of UHF is very necessary to analyze the propagation characteristics of UHF signals in switchgears. The GIS simulation model has been developed by the FDTD method for the research on propagation characteristics [13–16]. With a coaxial cavity structure, however, GIS has an entirely different structure from switchgears. The GIS conclusions may not apply to switchgear because their different structures. The transformer simulation model has also been developed for the study of propagation characteristics [17,18]. Transformer cores and windings play a decisive role in the propagation characteristics of transformers while the internal structure of switchgears is more complex and the two kinds of equipment can't be lumped together.

In this paper, an inner UHF antenna sensor based on the fractal technique has been developed. The sensor is designed in accordance with the features of UHF monitoring in switchgear. In addition, a switchgear simulation model has been developed by the FDTD method for the research into the propagation characteristics. The detection results of different monitoring points under various discharges have been analyzed by simulation and test methods, which offer the theoretical basis of the location of the inner sensor.

## 2. Inner UHF Antenna Sensor

The antenna is developed to be installed in the switchgear for the UHF monitoring method. Since the frequency of signals in air used in the UHF method are commonly distributed from 300 MHz to 1 GHz [9], and wide band information is necessary for such a method, the mentioned antenna should work over 300 MHz with wide band capability. However, as too much low-frequency noise exists, the antenna working frequency should be a little higher than 300 MHz to achieve better SNR. Besides, a fast data acquisition card would be used to transform the signal from the antenna into digital data for further analysis. With consideration of the UHF method requirements and economic factors, an antenna with a working band from about 500 MHz to 1 GHz would be developed.

The inner structure of switchgear is complicated, with a number of electric components, including the cabinet, partition boards, current transformers (CTs), circuit breakers (CBs), bushings, insulators, bus-bars, *etc.* CTs, CBs, insulators and bus-bars are the basic components installed in all types of switchgears. Due to the components within switchgears, there is not much space for an inner antenna. The space is further limited by the insulation requirements due to the air insulation used in switchgears. As a result, this places demands on the size and shape of the inner antenna. In addition, the inner antenna should also work with low standing wave ratio (VSWR), high gain and suitable directivity to collect PD signals inside the switchgear.

**Figure 1.**
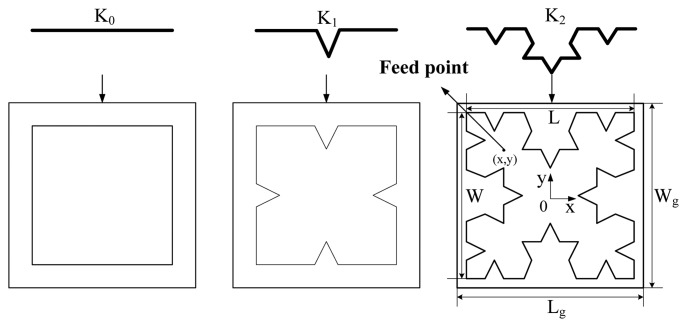
Koch fractal patch antennas with fractal dimensions of 0, 1 and 2.

To satisfy the above requirements, a micro-strip with a Koch structure will be developed as the inner antenna. The Koch curve is a type of fractal curve. This kind of fractal curve could make up the micro-strip antenna with Koch structure shown in Figure 1.

**Figure 2.**
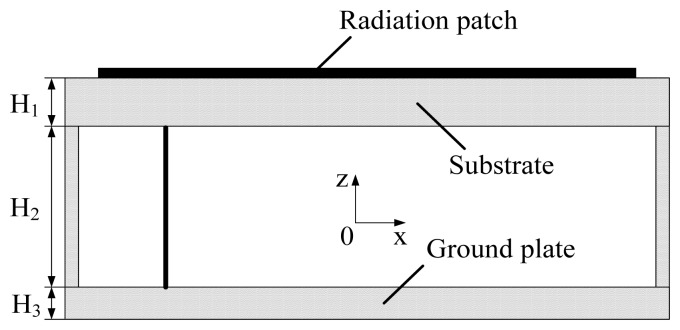
Patch antenna with an air layer structure.

The general micro-strip antenna commonly has a bandwidth of a few of percent, while the fractal structure could contribute to extend the frequency band [19]. Moreover, the adoption of a substrate with low permittivity is beneficial to spectral band extension. For this reason, as shown in Figure 2, the substrate and ground plate are separated by an air layer because of its low permittivity.

**Figure 3.**
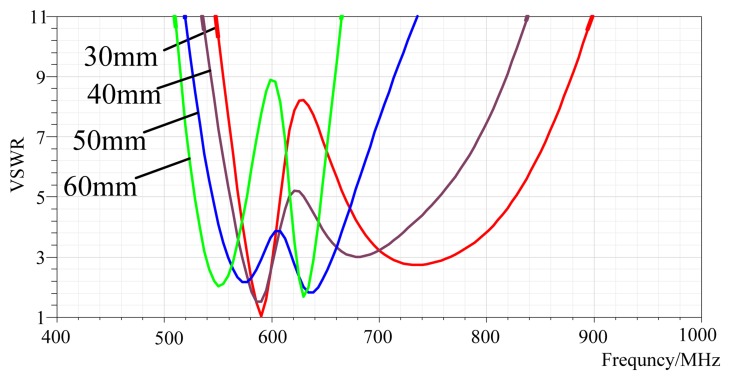
The VSWR curve of antennas with different feed point positions.

As shown in Figures 1 and 2, the structural parameters of the designed antenna consist of the dimensions of the radiation patch W and L, the feeding point coordinates (x,y), the relative permittivity of the substrate, the thickness of substrate H_1_, the dimensions of substrate W_g_ and L_g_ , the thickness of the air layer H_2_, the thickness of the ground plate H_3_ and the fractal dimension. The simulation model of the Koch fractal patch antenna has been developed using the High Frequency Structure Simulator (HFSS) software. Furthermore, the parameters above have been optimized with the optimization of the coordinated of the feeding point as an example shown in Figure 3. The feed point is located in the diagonal of the micro-strip, with its coordinate origin in the center of the micro-strip as seen in Figure 1.

**Figure 4.**
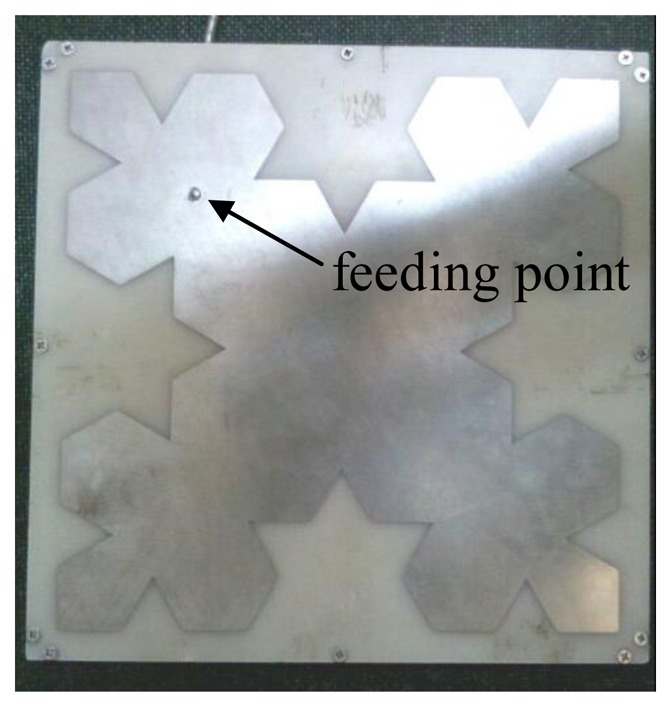
Picture of the designed antenna.

**Figure 5.**
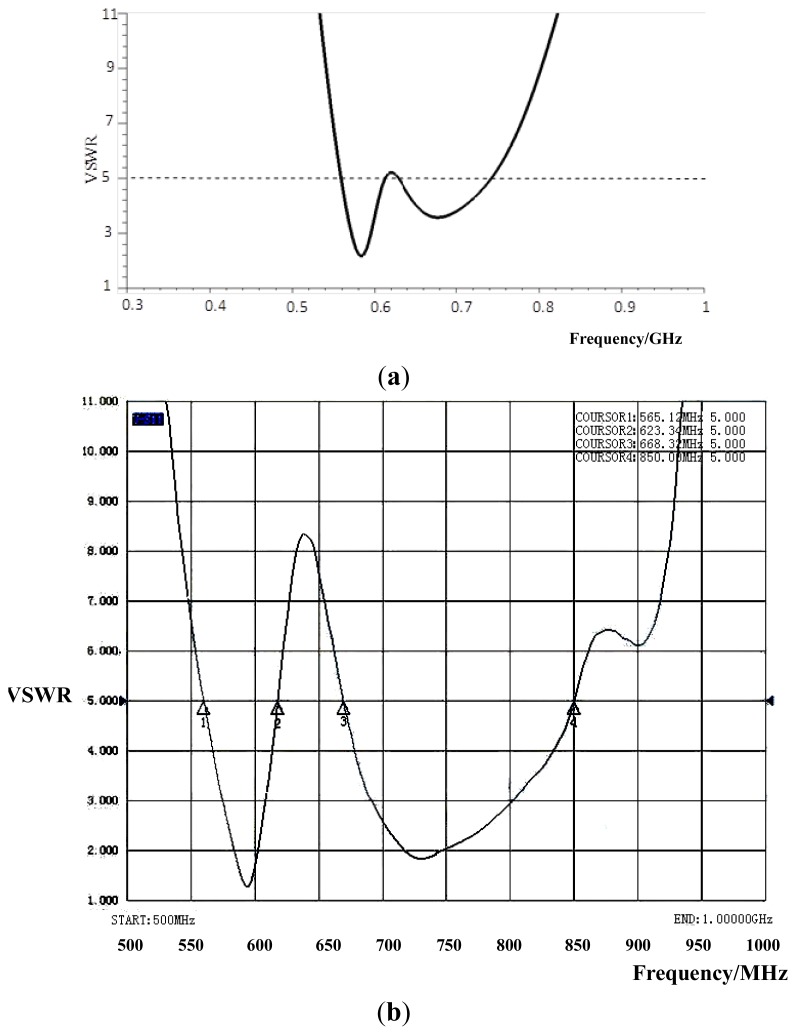
VSWR curve of the designed antenna. (**a**) VSWR curve in HFSS, (**b**) Measured VSWR curve.

The general parameters of the sensor are listed in Table 1. A picture of the designed antenna with a flat-plate structure is shown in Figure 4. Since the ground plate of antenna isn't the radiating element, the performance of this antenna is less affected by the switchgear cabinet when the sensor is installed on the inner side plate of the switchgear body. The VSWR curve, gain and directivity of the sensor have been measured by an AV3629 vector network analyzer, AV1487B signal source, HP8592L spectrum analyzer, and HP8720D vector network analyzer, respectively. The measured VSWR curve with two resonance frequencies is illustrated in Figure 5b. The first resonance frequency is 583 MHz with the VSWR of 1.3, the frequency band from 565 MHz to 623 MHz, the relative bandwidth of 10%, while the second one is 732 MHz with the VSWR of 1.7, the frequency band from 668 MHz to 850 MHz, the relative bandwidth of 24%. Compared with the VSWR curve in HFSS, the two curves are almost the same. Due to the construction, the peak between two resonance points of the measured curve is higher than the simulated one, while the overall bandwidth of the measured curve is better than the simulated one.

The gain of the sensor is listed in Table 2. The gain within the working band is over 1.4 dB, with which the sensor can detect weak PD signals. However, the gain is only −1.5 dB for 700 MHz because it's out of the working band. This indicates that signals outside the antenna working band will be attenuated too severely to be collected. Therefore, the low-frequency noise will be avoided efficiently.

**Figure 6.**
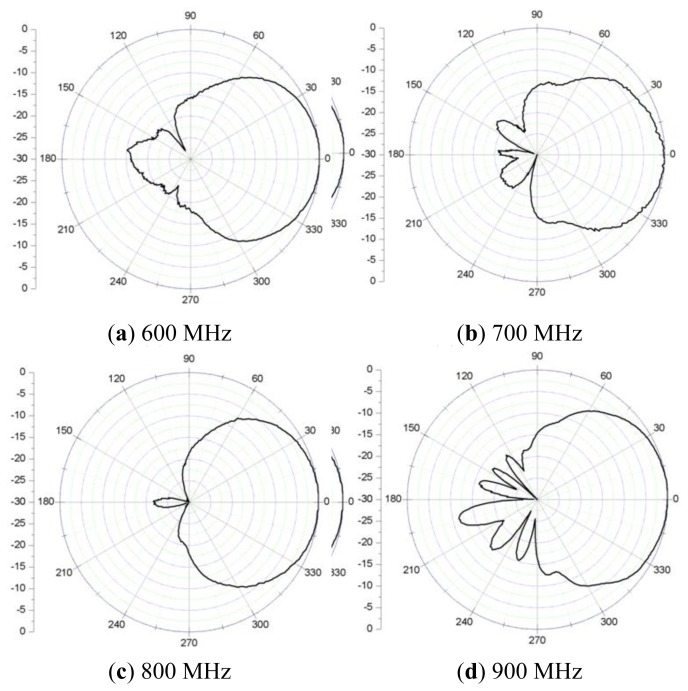
Measured E-plane radiation pattern of micro-strip antenna.

Compared to ultra-wide-band (UWB) antennas, the size of the developed antenna is smaller, which is more suitable for installation in switchgears. Its gain within the working band would be relatively higher due to deep VSWR curve in the two resonance frequency point. Last but not the least, as shown in Figure 6, the excellent vertical directivity of the antenna ensures that the signals above it can be collected best. In summary, the antenna satisfies the above requirements well.

**Figure 7.**
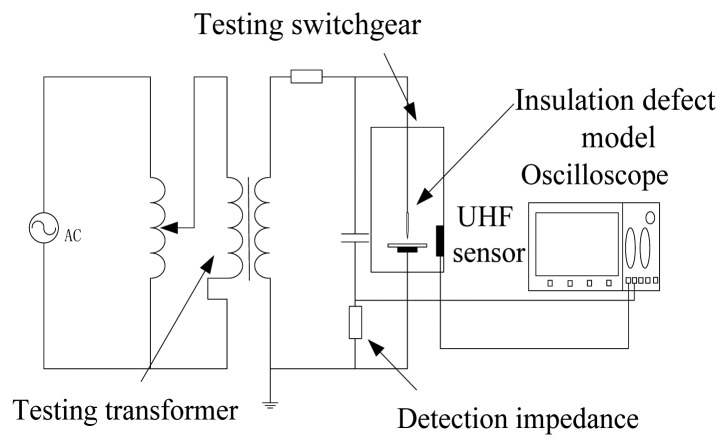
Experimental circuit.

**Figure 8.**
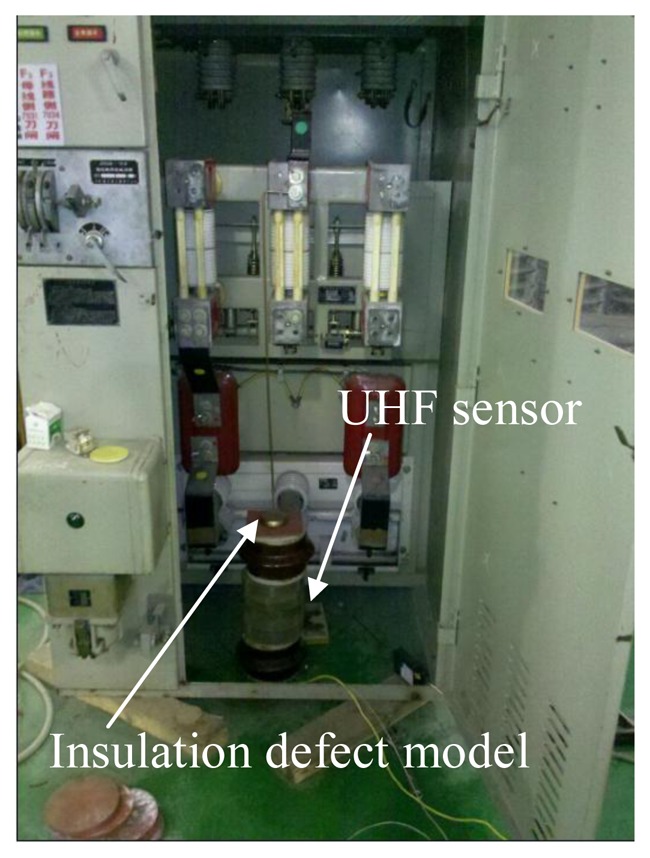
Testing switchgear.

Four kinds of insulation defect models have been developed in this paper, to simulate four main kinds of PDs by experiment. These insulation defect models are an air-gap insulation defect, surface flashover insulation defect, metallic outshoot insulation defect, and free metal particles insulation defect. The proposed sensor was investigated by experiment using the circuit illustrated in Figure 7. In the test, the detection impedance of the impulse current method was used as the reference sensor. The output signal was recorded using a Tektronix TDS 7104 oscilloscope with the band width of 1 GHz and a maximum sampling rate of 20 GS/s. The insulation defects and sensor were placed in a real switchgear cabinet as shown in Figure 8.

**Figure 9.**
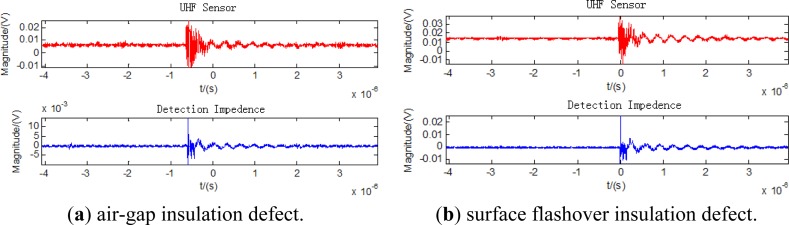

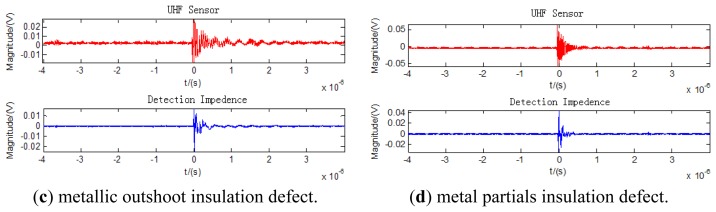
The measured waveforms of different kinds of discharges.

The measured waveforms of the air-gap, surface flashover, metallic outshoot, and metal particles insulation defects are illustrated in Figure 9a–d respectively. They show that the UHF sensor could reproduce the waveforms of the partial discharges of various kinds well.

## 3. Influence of Each Component in the Switchgear on the Propagation of Electromagnetic Waves

A switchgear has a quite complex internal structure, and each component of a switchgear has different effects on the propagation of UHF signals. Hence, the emphasis of this section is placed on the influence of the actual size and structure of the switchgear on the propagation of radio waves.

**Figure 10.**
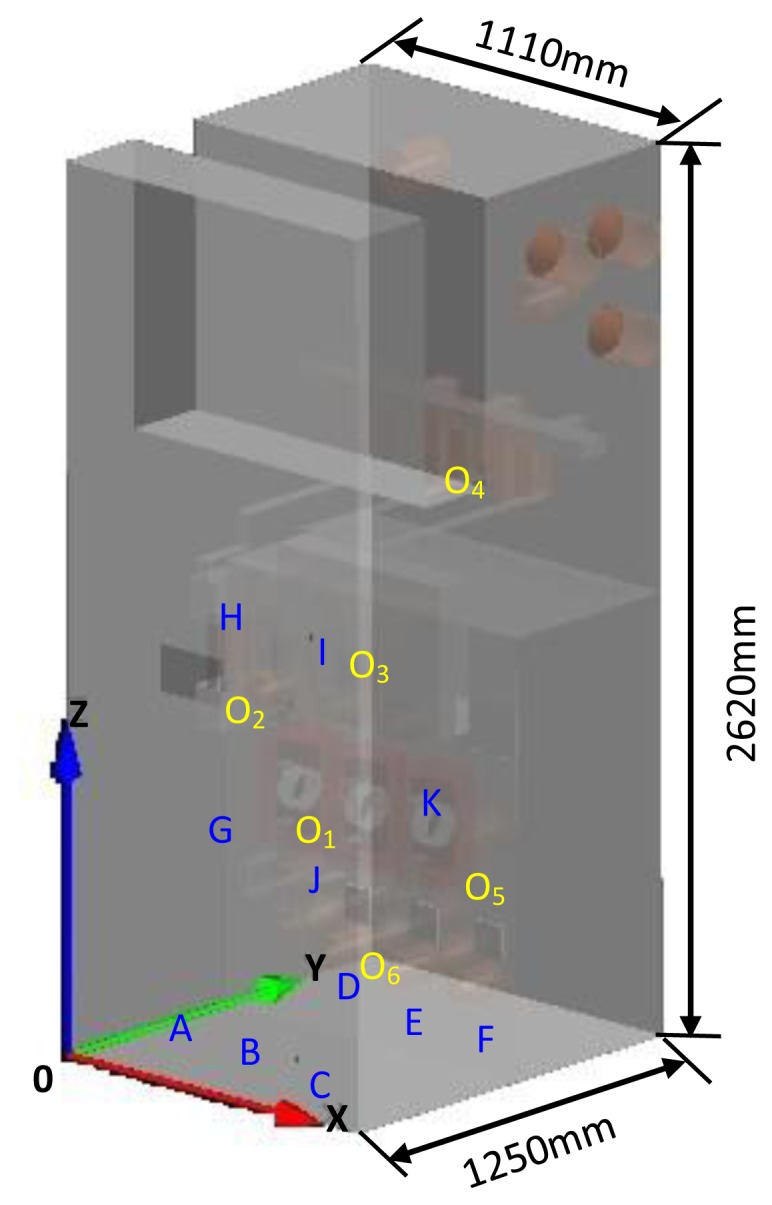
FDTD switchgear simulation model.

Figure 10 shows an FDTD simulation model of a switchgear with the dimensions of 1,110 mm × 1,250 mm × 2,620 mm that consists of a cabinet, partition boards, CTs, CBs, bushings, insulators and bus-bars. The material parameters of each component are listed in Table 3. The FDTD method is based on mesh generation, and the meshing size closely relates to the accuracy of the result and the amount of calculation. To balance calculation time and precision, the meshing size should be appropriate. According to the Courant condition, the maximum mesh size L_max_ should be calculated as [20]:

(1) $$L_{\max}=\frac{c}{10\times f}$$

where *c* is the speed of light and *f* is the maximum frequency of the excitation source. The maximum frequency of our simulation analysis was set at 3 GHz due to the UHF band being used. Therefore, according to Equation (1), the maximum mesh size L_max_ was 10 mm. In this article, the simulation model was meshed uniformly in a three-dimensional system. Then the corresponding time step Δt was calculated to be 19.26 ps. It must be mentioned that the simulation boundary condition is determined to be a perfectly matched layer (PML). The partial discharge source used in the simulation was a Gaussian pulse with broad frequency characteristics as defined in Equation (2):

(2) $$I(t)=I_{0}\exp(-\frac{4\pi {\left(t-t_{0}\right)}^{2}}{\tau^{2}})$$

where *τ* is a constant that determines the width of the pulse waveform, *t*_0_ is the time when the peak waveform occurs and *I*_0_ is the amplitude of the waveform. The waveform was determined to have *τ* of 616 ps, *t*_0_ of 616 ps and *I*_0_ of 1 A. The discharge capacity of the Gaussian pulse was calculated to be 359.6 pC by waveform integration.

### 3.1. Influence of CT on the Propagation of EM Waves

**Figure 11.**
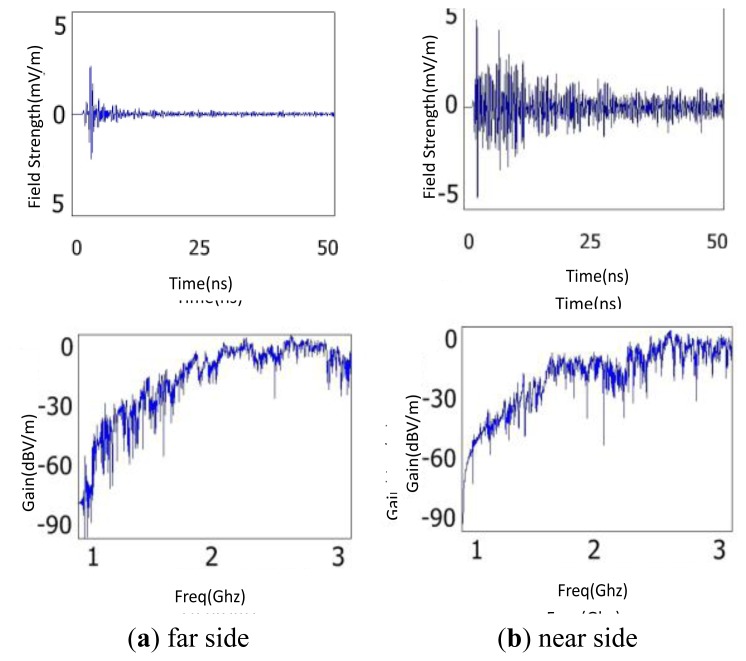
Simulation results of the influence of CT on the propagation of EM waves.

The size of CT with the iron core structure and windings is relatively larger than the other components, so the influence of the component on the propagation of EM waves can't be ignored. The proposed discharge source was placed near the B-phase CT. Two detection points were located on the near and far sides of the CT relative to the discharge source, respectively. Figure 11a,b is the simulation results of the near and far sides, respectively.

It can be seen from the comparison of the time domain waveforms that the waveform amplitude reduces by as much as 6 dB when the EM waves pass through the CT. In addition, the amplitude dwindles rapidly by more than 20 dB after the first peak appears. The Fast Fourier Transform (FFT) waveforms show that the CT makes the low frequency components (the frequency band below 1.5 GHz, the same below) attenuate greatly while the high frequency ones (the frequency band above 1.5 GHz, the same below) hardly change. According to these results, the sensor should not be positioned near CTs due to their influence of attenuation of EM waves.

### 3.2. Influence of CB on the Propagation of EM Waves

**Figure 12.**
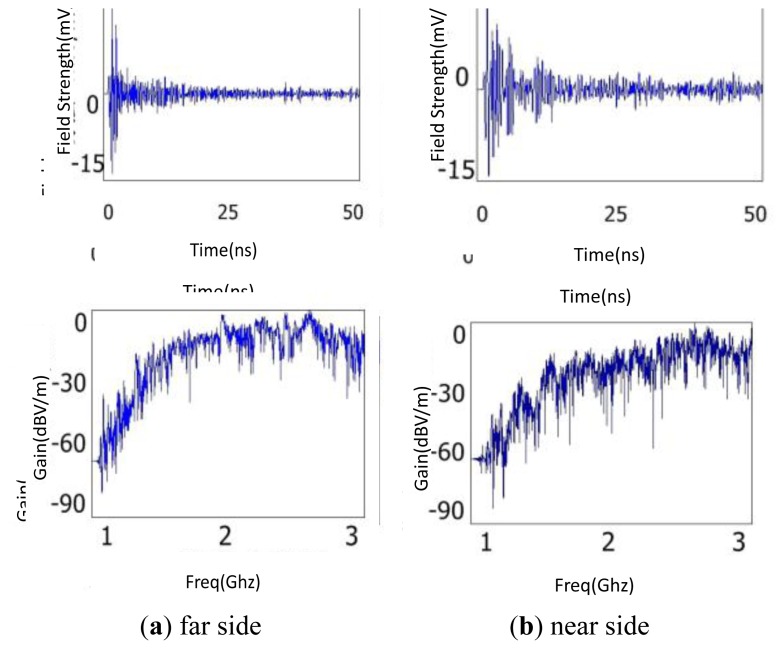
Simulation results of the influence of CB on the propagation of EM waves.

CB is the most important part of switchgear, and is normally located in the breaker chamber. The CB of the simulation model is a vacuum circuit breaker with a porcelain vase structure outside and a metal shield shell inside. The pulsed discharge signal was placed near the B-phase CB. Two detection points were located on the near and far sides of the CB relative to the discharge source, respectively. Simulation results are shown in Figure 12a,b. From the comparison of the time domain waveforms, the radio waves spread through the CB with little attenuation. It is likely that although the EM waves pass into the porcelain vase with a little dielectric attenuation, they diffract with little decay when meeting the metal shield shell as the size of the shell is rather smaller than that of electric waves. It's worth noting that the waveform will distort thanks to the existence of CB. The FFT waveforms also indicate that CB has a limited effect on the spectrum distribution. From these simulation results, it is concluded that the installation position of the sensor does not have to avoid the CB areas under the condition that the small waveform distortion can be neglected.

### 3.3. Influence of the Insulator on the Propagation of EM Waves

**Figure 13.**
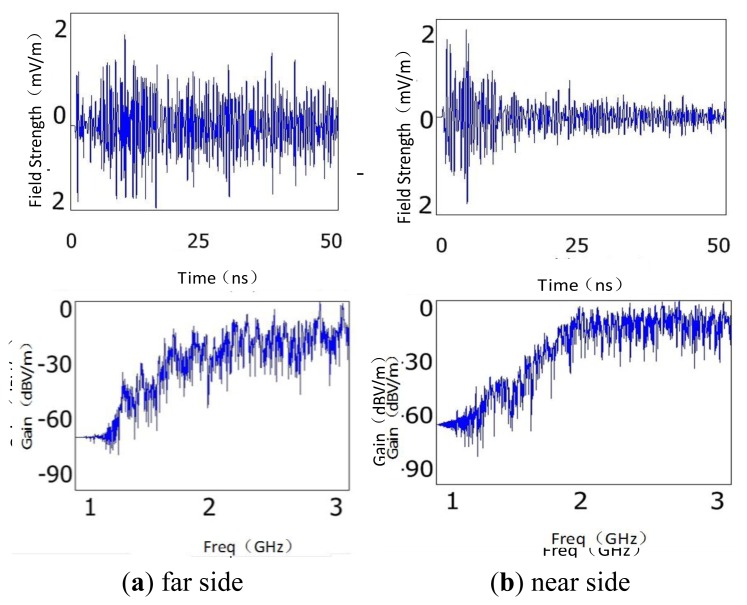
Simulation results of the influence of insulator on the propagation of EM waves.

Since there is a large quantity of insulators in switchgear, EM waves inevitably spread into the regions of these insulators. The artificial discharge source was placed near an insulator. Then two detection points were placed on the near and far sides of the insulator relative to the discharge source, respectively. By comparing the measured waveforms illustrated in Figure 13a,b, we see that the insulator affects the waveform amplitude slightly, but with great wave shape distortion. It is shown from the FFT waveforms, the low frequency components are weakened by the insulator. It can be concluded that the UHF antenna should not be located next to insulators because the severe wave distortion would affect pattern recognition.

### 3.4. Influence of Bus-Bar on the Propagation of EM Waves

**Figure 14.**
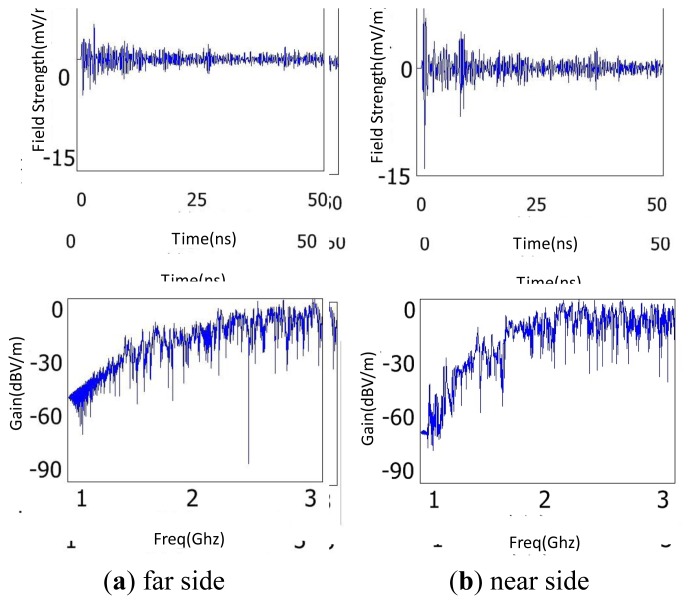
Simulation results of the influence of bus-bar on the propagation of EM waves.

The metal-material bus-bars are widely distributed in the cabinet, so the influence of bus-bars on the propagation of EM waves cannot be ignored. The above discharge source was located near the bus-bar connected with the B-phase CT. Then two detection points were placed on the near and far sides of the insulator relative to the discharge source, respectively. Figure 14a,b is the simulation results of the near and far sides, respectively. Through the time domain waveforms, it is shown that the electric wave decays by 3.5 dB because of the reflection losses. Then the FFT waveforms indicate that the high frequency components are restrained by the bus-bar. From these results, the sensor should not be positioned near bus-bars because they will be disadvantageous to signal reception.

## 4. The Optimal Installation Position of the Inner Sensor

According to the results of Section 3, it can be found that the components in the switchgear affect the electromagnetic waves differently and they affect electromagnetic waves as a whole. As one of the most important components in the UHF monitoring system, the UHF sensor should be installed in an appropriate place to optimize detection results. In this section, the best place to position the inner sensor will be studied.

### 4.1. Simulation Analysis

According to the simulation analyses of Section 3, in this article, several detection points were set in the simulation model. Then, referring to the common types and locations of discharges that occur in switchgear in [21], various discharge sources were placed. The waveform amplitudes would be used to determine the best detection point, namely, the optimal installation position of the inner sensor. Moreover, the mentioned discharge source is the same as in Section 3.1. The locations of discharge sources and detection points are shown in Figure 10 and the coordinates are listed in Table 4.

The amplitudes of electric field strength in each detection point are listed in Table 5. In order to facilitate comparison of results, in Table 5 each value is divided by the maximum value in each row for normalization. Then the processed values of each column were added up to a total value, which is related to the overall detection effect of the corresponding detection point. Furthermore, the larger the value is, the better the overall detection effect is. The variance of values in each column was also figured out to determine the volatility of the detection for different discharge sources. The smaller the variance is, the less the volatility is. All the processed data was listed in Table 6. In general, the detection point C located on the right side of the bottom plate and the detection point I located on the bottom-right side of the front cupboard door have good satisfied detection performance with overall consideration. From the viewpoint of safety, only the detection point I is close to CTs, which probably brings a new insulation fault hazard. Moreover, if the detection point I is selected for the sensor, the sensor need to be anchored vertically with more difficulty than point C, so in conclusion, the inner sensor should be positioned in the point C for the best detection performance.

### 4.2. Experimental Verification

According to the results in Table 6, the detection performance of each detection point located on the same mounting surface obviously don't vary from each other, so there is no necessity to verify the detection performance of every point. As a result, the four detection points with the best detection performance in each mounting surface were chosen to be the experimental verification points. They are point C, point F, point I and point K (marked in gray).The wiring diagram is illustrated in Figure 7, and the components of this circuit are the same as those in Section 2. It must be mentioned that in each test, the values of the voltage on the same insulation defect model and the discharge capacity of discharge (*i.e.*, waveform amplitudes got by the detection impedance) in the same defect model should be equal to each other. The test data can be collected ten minutes after the voltage was applied. Furthermore, each case contained 10 tests, and the average of these results was taken as the final result in order to reduce the random error as much as possible.

**Figure 15.**
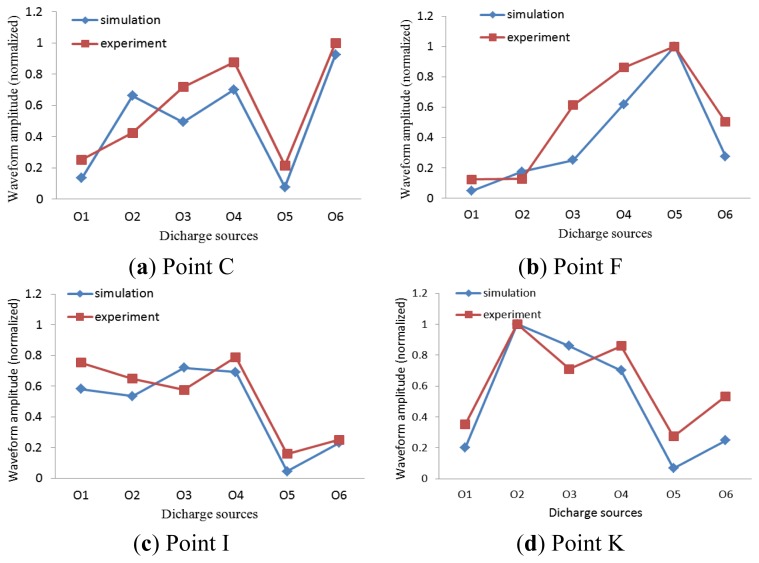
Comparison between the results of test verification and simulation computation.

The comparison between the results of test verification and simulation computation is shown in Figure 15. According to these Figures, it can be seen that, on the condition of the discharge O_3_, the error of the two methods is relatively larger than that under other conditions, because the discharge O_3_ is a kind of free metal particles PD discharge. It's a sort of non-stationary discharge. The metal particles would move irregularly in an electric field, which makes the strength of the discharge change greatly throughout the experiment. In general, errors occurred under most conditions. However, the simulated curves agree well with those of experiments. In other words, the simulation analysis method of is correct and feasible. Besides, the results indicate that the antenna with vertical directivity is best suitable for the location point C. Since the antenna would be installed at point C, the PD signal above it can be directly collected by the antenna, which best avoids the distortion and attenuation due to catadioptric effects and it ensures the integrity of the signals for better analysis.

## 5. Conclusions

An inner UHF fractal antenna sensor which was specially designed for PD detection in switchgear was developed. The UHF sensor had two frequency bands. The first resonance frequency is 583 MHz with the VSWR of 1.3, the frequency band from 565 MHz to 623 MHz, the relative bandwidth of 10%, while the second one is 732 MHz with the VSWR of 1.7, the frequency band from 668 MHz to 850 MHz, the relative bandwidth of 24%. The waveforms of different kind of PDs in the switchgear can be measured with a high sensitivity.

In order to determine the optimal installation position of the inner senor, the FDTD simulation model was developed to study the propagation characteristics of UHF signals in switchgears. The simulation indicated that CTs and bus-bars would make the EM waves attenuate greatly and insulators would affect the EM waves with obvious distortion of the wave shape. The sensor should not be positioned near these three kinds of components. CBs had little influence on the radio waves, so the installation position of sensor may not avoid their areas. According to simulation analyses and experimental verification, the UHF sensor should be positioned at the right side of the bottom plate in the front cabinet (*i.e.*, breaker chamber) to achieve the best detection performance.

## Figures and Tables

**Table 1.** Dimensions of the microstrip antenna (in mm).

**Fractal Dimension**	**W**	**L**	**Wg**	**Lg**	**H1**	**H2**	**H3**	**Relative Permittivity**	**Feeding Point**
2	170	170	180	180	5	20	1	4.2	(40.5,40.5)

**Table 2.** Measured gain of the microstrip antenna.

**Frequency (MHz)**	600	700	800	900

**Gain (dB)**	3	−1.5	1.4	1.7

**Table 3.** The material parameters of each component.

**Each Component of the Switchgear**	**Relative Permittivity (ε_r_)**	**Relative Permeability (μ)**	**Electrical Conductivity σ (S/m)**
Cabinet	1	-	1.1 × 10^6^
Copper bus-bar	1	-	5.7 × 10^7^
Umbrella skirt of insulator	5.7	-	1 × 10^−8^
Pouring material of CT	3.6	-	1 × 10^−8^
Iron core of CT	-	2,000	-
Porcelain vase of break	1	-	6.5 × 10^7^

**Table 4.** Coordinates of discharge sources and detection points.

	**Locations**		**Coordinates**
Discharge sources	Inside the B-phase CT (O_1_)		(440,510,590)
	On the surface of the supporting insulator of the A-phase CB (O_2_)		(440,440,1060)
	Around the C-phase CB (O_3_)		(770,330,1210)
	On the surface of the bushing at the upside of back cabinet (O_4_)		(390,855,1910)
	Inside cable joint at the downside of back cabinet (O_5_)		(580,780,290)
	On the surface of the bushing at the downside of front cabinet (O_6_)		(630,520,280)

Detection points	Bottom plate of the front cabinet	Left side (A)	(150,340,0)
		Middle side (B)	(520,340,0)
		Right side (C)	(770,340,0)

	Bottom plate of the back cabinet	Left side (D)	(150,940,0)
		Middle side (E)	(520,940,0)
		Right side (F)	(770,940,0)

	Front cupboard door	Bottom-left side (G)	(520,0,580)
		Upper-left side (H)	(520,0,1500)
		Bottom-right side (I)	(770,0,580)
		Upper-right side (J)	(770,0,580)

	Side plate of the front cabinet	Middle side (K)	(1110,340,600)

**Table 5.** Amplitude field strength of each detection point.

**Point**	**Amplitude Field Strength of Each Detection Point (mV/m)**					

	O_1_	O_2_	O_3_	O_4_	O_5_	O_6_
A	2.61	4.87	1.98	2.81	1.87	1.47
B	2.65	4.62	2.49	2.34	1.27	6.73
C	2.37	5.53	2.37	3.12	1.37	6.24
D	0.72	1.34	1.21	0.72	7.12	1.84
E	0.89	1.12	1.22	0.56	15.75	1.62
F	0.83	1.47	1.2	0.62	17.74	1.86
G	17.46	4.12	1.72	1.86	0.81	3.14
H	2.22	4.46	2.49	4.45	0.42	1.55
I	10.16	4.46	3.45	3.08	0.82	3.14
J	2.34	4.23	4.79	3.87	0.38	1.43
K	3.52	8.34	4.12	3.12	1.23	1.67

**Table 6.** Amplitude field strength of each detection point (normalized).

**Point**	**Amplitude Field Strength of Each Detection Point (Normalized)**							

	O_1_	O_2_	O_3_	O_4_	O_5_	O_6_	Total	Variance
A	0.149	0.12	0.413	0.631	0.105	0.218	1.636	0.044
B	0.146	0.554	0.52	0.526	0.072	1	2.818	0.111
C	0.136	0.663	0.495	0.701	0.077	0.927	2.999	0.112
D	0.041	0.161	0.253	0.72	0.401	0.273	1.849	0.055
E	0.051	0.134	0.255	0.56	0.888	0.241	2.129	0.098
F	0.048	0.176	0.251	0.62	1	0.276	2.371	0.124
G	1	0.494	0.359	0.418	0.046	0.467	2.784	0.095
H	0.127	0.511	0.52	1	0.024	0.23	2.412	0.126
I	0.582	0.535	0.72	0.692	0.046	0.23	2.805	0.073
J	0.134	0.507	1	0.87	0.021	0.212	2.744	0.164
K	0.202	1	0.86	0.701	0.069	0.248	3.08	0.151

## References

[1] Portugues I.E., Moore P.J., Glover I.A., Johnstone C., McKosky R.H., Goff M.B., van der Zel L. (2009). RF-based partial discharge early warning system for air-insulated substations. IEEE Trans. Power Del..

[2] Coenen S., Tenbohlen S., Markalous S.M., Strehl T. (2008). Sensitivity of UHF PD measurements in power transformers. IEEE Trans. Dielectr. Electr. Insul..

[3] Hikita M., Okabe S., Murase H. (2008). Cross-equipment evaluation of partial discharge measurement and diagnosis techniques in electric power apparatus for transmission and distribution. IEEE Trans. Dielectr. Electr. Insul..

[4] Kweon D.J., Chin S.B., Kwak H.R. (2005). The analysis of ultrasonic signals by partial discharge and noise from thetransformer. IEEE Trans. Power Del..

[5] Chen L.J., Tsao T.P., Lin Y.H. (2005). New diagnosis approach to epoxy resin transformer partial discharge using acoustic technology. IEEE Trans. Power Del..

[6] Li D.J., Liang J.Z., Bu K.W., Yang J.G., Li Y.M. (2009). Ultrasonic detection of partial discharge on typical defects in GIS (in Chinese). High Volt. Appar..

[7] Guan Y.G., Qian J.L. (2007). Practical study of radio frequency signal used in on-line PD monitoring of hv switchboard (in Chinese). High Volt. Appar..

[8] Ren M., Dong M., Ren Z., Peng H.D., Qiu A.C. (2012). Transient earth voltage measurement in PD detection of artificial defect models in SF6. IEEE Trans. Plasma Sci..

[9] Tenbohlen S., Denissov D., Hoek S.M., Markalous S.M. (2008). Partial discharge measurement in the ultra high frequency (UHF) range. IEEE Trans. Dielectr. Electr. Insul..

[10] Li T.H., Rong M.Z., Zheng C., Wang X.H. (2012). Development simulation and experiment study on UHF partial discharge sensor in GIS. IEEE Trans. Dielectr. Electr. Insul..

[11] Hoshino T., Kato K., Hayakawwa N. (2001). A novel technique for detecting electromagnetic wave caused by partial discharge in GIS. IEEE Trans. Power Del..

[12] Tang Z.G., Li C.R., Cheng X., Wang W., Li J.Z., Li J. (2006). Partial discharge location in power transformers using wideband RF detection. IEEE Trans. Dielectr. Electr. Insul..

[13] Hikita M., Ohtsuka S., Teshima T., Okabe S., Kaneko S. (2007). Examination of electromagnetic mode propagation characteristics in straight and L-section GIS model using FD-TD analysis. IEEE Trans. Dielectr. Electr. Insul..

[14] Hoshino T., Maruyama S., Sakakibara T. (2009). Simulation of propagating electromagnetic wave due to partial discharge in GIS using FDTD. IEEE Trans. Power Del..

[15] Hu X., Judd M.D., Siew W.H. A Study of PD Location Issues in GIS using FDTD Simulation.

[16] Li X., Li C.R., Li Y.S., Wang W., Li H.L. (2005). Analysis on partial discharge in GIS by FDTD method. Proc. CSEE.

[17] Judd M.D., Li Y., Hunter B.B.I. (2005). Partial discharge monitoring for power transformers using UHF sensors part I: Sensors and signal interpretation. IEEE Electr. Insul. Mag..

[18] Aung M.T., Milanvic J.V. (2006). The Influence of transformer winding connections on the propagation of voltage sags. IEEE Trans. Power Del..

[19] Puente-Baliarda C., Romeu J., Pous R., Cardama A. (1998). On the bahavior of the Sierpinski multiband fractal antenna. IEEE Trans. Antennas Propag..

[20] Lee J.H., Kalluri D.K. (1999). Three-dimensional FDTD simulation of electromagnetic wave transformation in a dynamic inhomogeneous magnetized plasma. IEEE Trans. Antennas Propag..

[21] Wang F.L. (2009). Well-Chosen Cases of Applications of Condition Monitoring in Electrical Equipment (in Chinese)..

